# Psychological treatments for common mental health problems experienced by informal carers of adults with chronic physical health conditions (Protocol)

**DOI:** 10.1186/2046-4053-2-9

**Published:** 2013-01-31

**Authors:** Joanne Woodford, Paul Farrand, David Richards, David J Llewellyn

**Affiliations:** 1 Mood Disorders Centre, Psychology, College of Life and Environmental Sciences, University of Exeter, Exeter, UK; 2 University of Exeter Medical School, Exeter, UK

**Keywords:** Caregivers, Chronic physical health condition, Depression, Anxiety, Treatment, Systematic review

## Abstract

**Background:**

Improved life expectancy is resulting in increased outpatient treatment of people with chronic physical health conditions and reliance on the provision of informal care in the community. However, informal care is also associated with increased risk of experiencing common mental health difficulties such as depression and anxiety. Currently there is a lack of evidence-based treatments for such difficulties, resulting in poor health outcomes for both the informal carer and care recipient.

**Methods/Design:**

Electronic databases will be systemically searched for randomised controlled trials examining the effectiveness of psychological interventions targeted at treating depression or anxiety experienced by informal carers of patients with chronic physical health conditions. Database searches will be supplemented by contact with experts, reference and citation checking and grey literature. Both published and unpublished research in English language will be reviewed with no limitations on year or source. Individual, group and patient-carer dyad focused interventions will be eligible. Primary outcomes of interest will be validated self-report or clinician administered measures of depression or anxiety. If data allows a meta-analysis will examine: (1) the overall effectiveness of psychological interventions in relation to outcomes of depression or anxiety; (2) intervention components associated with effectiveness.

**Discussion:**

This review will provide evidence on the effectiveness of psychological interventions for depression and anxiety experienced by informal carers of patients with chronic physical health conditions. In addition, it will examine intervention components associated with effectiveness. Results will inform the design and development of a psychological intervention for carers of people with chronic physical health conditions experiencing depression and anxiety.

PROSPERO registration number: CRD42012003114

## Background

Advances in public health and medical technology have resulted in continued increases in life expectancy across developed countries [1]. Within the UK alone the projected rise in adults aged over 80 years is due to rise from 2.9 million adults in 2010 to 5.9 million in 2035 [2]. These increases in life expectancy are presenting significant challenges to existing healthcare systems with regards to the management and treatment of patients with chronic or disabling illnesses [1,3]. This is manifesting itself in an increased reliance on informal carers as a fundamental part of patient management which has become important following an increasing emphasis upon outpatient treatment of patients with chronic physical health conditions [4]. Currently around 5 million people in the UK provide informal care to someone with a physical or mental health difficulty [5].

The shift to outpatient treatment alongside a concomitant increase in the role of informal carer in patient management and treatment has led to a reduction in patient hospital and physician care as well as delaying the receipt of nursing home care [6]. However given demands associated with supporting the treatment and recovery of patients with a physical or mental health difficulty now being placed upon informal carers, the potential is that costs are simply being shifted elsewhere. Informal care is not only associated with greater risks of poor mental and physical health [7-10] but additional personal and societal costs arising from reductions in hours of paid work, restriction in social and recreational activities [11], and sleep disturbances [12]. Additionally poor mental health in carers may also negatively impact on outcomes associated with the care recipient [13].

A clear need therefore exists to develop evidence-based psychological interventions to support the long-term emotional needs of informal carers. However such long-term emotional needs of carers have been neglected across a range of chronic physical health conditions [14-18]. Furthermore, services that do exist to provide emotional support are often inadequately developed and are generally not tailored to address the unique difficulties carers’ experience [19]. Such difficulties may include the management of behavioural problems [7], physical impairments [20], cognitive decline [21] and the development of communication techniques [22]. Developing interventions have however potentially been hindered given that the needs of carers often change dependent upon the course of the chronic physical health condition of the care recipient, the setting care is provided in and length of time care has been provided [17]. Recognition of the unique and multifaceted needs of informal carers has led to the suggestion that multicomponent interventions are required [17,23].

A number of meta-analyses have been undertaken to identify factors associated with positive outcomes in informal carers, such as caregiver burden, knowledge, depression and symptoms of care recipients. Commonly such meta-analyses have included a large number of potential factors, such as respite and day care, knowledge and training, group- and individual-based interventions, type of setting and various caregiver characteristics [24]. Additionally they have focused upon a variety of specific patient carer groups such as stroke [25], cancer [26] and dementia [23,27]. Far less attention has however been directed towards identifying specific psychological interventions that may be targeted at the treatment of depression and anxiety. Where this focus has been included within the meta-analysis as treatment moderators, psychological treatments mostly consistent with elements of cognitive behavioural therapy (CBT) have been identified to have a small effect on caregiver depression [24,28]. Furthermore, one review has identified cognitive reframing, a specific component commonly found within CBT, as an intervention component promising for depressed informal carers of dementia patients [21].

To date little research has sought to identify specific intervention components that have been utilised in interventions targeting depression and anxiety in carers of adults with chronic physical health conditions. Additionally, when such components have been identified through approaches such as systematic reviews, it has been reported that too little attention has then been paid to identifying the specific components associated with effectiveness [29,30]. Recent systematic reviews have therefore examined not only the overall effectiveness of interventions but also the specific intervention components associated with their effectiveness [30,31]. This systematic review therefore seeks to examine both the overall effectiveness of psychological interventions for depressed or anxious carers and specific intervention components associated with effectiveness. The identification of effective intervention components utilised in interventions targeting depression and anxiety in carers of adults with chronic physical health conditions is an important next step to inform the future design and development of evidence-based treatments.

### Objectives

First, to undertake a comprehensive systematic review and meta-analysis examining the effectiveness of psychological interventions targeted at treating emotional difficulties, such as depression or anxiety, across a range of carer-care recipient populations. Second, to identify intervention components associated with effectiveness. The results of the systematic review will also be used to feed into the development of an evidence-based complex intervention for carers of people with chronic physical health conditions using the Medical Research Council’s (MRC) guidance [32,33].

## Methods

The review will follow the Centre for Reviews and Dissemination (CRD) guidance on undertaking systemic reviews [34] and be reported to established criteria [35]. The review is registered with the PROSPERO International Prospective Register of Systematic Reviews (registration number CRD42012003114).

### Inclusion and exclusion criteria

#### Population

Eligible populations are informal adult (aged 16 years and older) carers of adults with chronic physical health conditions who are experiencing depression or anxiety. Formal diagnosis of depression or anxiety will not be required. No limitations will be placed on severity of depression or anxiety (though it is estimated carers will be depressed or anxious), length of time caring, chronic physical health condition of the person cared for, or relationship to person cared for. Informal carers will be defined as non-professionals who support people who are sick, infirm or disabled [36]. Commonly this group is made up of the patients’ close family, however non-family informal carers will be eligible for inclusion. Given the recognition that provision of care is dynamic and fluctuates from providing intensive assistance on a daily basis to more infrequent support no constraints will be placed on how much assistance informal carers provide [37].

#### Interventions

The review will include psychological or psychosocial interventions that are targeted at depression or anxiety. There will be no limitation in terms of psychological theory informing the intervention, the person delivering the intervention or the setting in which the intervention is delivered. Group, one-to-one and unsupported interventions will be included. Interventions for the carer-patient dyad will also be included as long as a target of the intervention is carer depression or anxiety.

#### Comparators

Only interventions compared with an inactive control will be considered. This may include: a waiting list control; treatment-as-usual (normally defined as standard care provided by a general practitioner/family doctor); no treatment and attention-controls. Interventions compared with another active intervention will not be eligible for inclusion.

#### Outcomes

Studies eligible for inclusion will have a primary or secondary outcome measurement of a validated self-report or clinician administered measure of depression or anxiety that elicits continuous data. Outcomes of caregiver burden and quality of life will also be examined. Dropout rates will also be recorded. Outcomes for any time period will be eligible for inclusion. However, in the case of studies reporting multiple time points the follow-up time point used for analysis will be the longest time point ≤6 months.

#### Study design

Only randomised controlled trials using a method of random sequence generation and allocation concealment assessed as low or unclear risk of bias using the Cochrane Collaboration’s Risk of Bias tool [38] will be included within the review.

### Search strategy

A comprehensive search will be conducted on the following electronic databases: Cumulative Index to Nursing and Allied Health Literature (CINAHL); Excerpta Medica DataBase (EMBASE); PsychInfo; Medline; Social Science Citation Index; Applied Social Sciences Index and Abstracts (ASSIA) and the Cochrane Central Register of Controlled Trials (CENTRAL). Reference lists and citations will be hand searched for all included studies to identify further studies. The results of the database searches will be analysed to identify journals that contain the largest number of included studies which will be hand searched for recent publications and conference abstracts (less than 12 months). Trial registers http://www.ClinicalTrials.gov and http://www.who.int/trialsearch/ will also be searched to identify on-going or unpublished trials. Experts in the field will be contacted to further identify unpublished or ongoing trials. An information specialist was consulted to build the search strategy using medical subject headings (MeSH). The Ovid MEDLINE search strategy can be found in Additional file 1.

### Study selection

All titles and abstracts will be screened by JW and a second researcher. Full paper review to determine inclusion will be conducted independently by JW and PF. Cohen’s Kappa will be calculated to determine agreement in selecting studies in accordance with the exclusion / inclusion criteria. Any discrepancies will be resolved by discussion and, if consensus cannot be reached, a third member of the research team will make the final decision.

### Data extraction

Data extraction will be conducted by JW and a researcher not associated with the research team. Discrepancies will be discussed and if consensus is not reached discussion will be held with PF. A data extraction form specifically for this review has been developed upon guidance from the CRD [34].

To meet the second objective of the review there will also be a specific focus on extracting information relating to intervention components and patient characteristics in addition to the standard extraction of information (for example, identification features, study characteristics, primary outcome measurements, statistical approaches and primary results). Intervention components extracted from the data are partially based upon those used in a previous review examining intervention components associated with increased effectiveness in diet and physical activity interventions [31]. Specifically the following will be extracted: (1) theoretical framework (for example, cognitive therapy, behaviour therapy, interpersonal therapy, psychodynamic therapy); (2) behaviour change techniques (for example, problem solving, goal setting, relapse prevention) based on a taxonomy of 137 behaviour change techniques [39]; (3) mode of delivery (for example, individual face-to-face, telephone, email, group, unsupported self-help); (4) group size for group-based interventions; (5) clinician delivering treatment (for example, nurse, general practitioners, clinical psychologist); (6) training received by the clinicians delivering the treatment; (7) treatment intensity (for example, duration of treatment, number of sessions, length of sessions); (8) whether the treatment is manualised (yes or no); (9) measurement of treatment integrity (yes or no); and (10) treatment setting (for example, primary care, secondary care). In addition, specific characteristics will be extracted for both the carer (for example, age, ethnicity, severity of depression or anxiety at baseline, length of time caring, relationship to person cared for and receipt of formal care in the home) and the adult with the chronic physical health condition (for example, age, chronic physical health condition, severity of chronic physical health condition and mental health and other chronic physical health co-morbidities). The data extraction form can be found in Additional file 2.

Intervention components will be extracted from published papers however all authors will also be contacted to obtain trial protocols and treatment manuals associated with the delivery of the intervention to enable more detailed coding to take place. Interventions will be coded by JW and PF independently and discrepancies will be resolved through discussion and if required a third researcher will be consulted.

### Methodological quality

The Cochrane Collaboration’s Risk of Bias tool [38] will be adopted to appraise the methodological quality of the included studies. This will be undertaken independently by JW and a reviewer not associated with the research team. Ratings will be compared and any discrepancies discussed, and if consensus is not reached, further discussion will be held with PF. The tool will examine risk of selection, performance, attrition and reporting bias. To detect reporting bias attempts will be made to obtain study protocols for all included studies either via published protocols, trial databases or emailing the study authors. Comparisons will be made with the outcome measurements reported in the protocol and the paper. In addition, outcomes reported in the methods section will be compared with outcomes reported in the results section. In the event of discrepancies study authors will be contacted to identify potential reasons, such as changes to the study protocol, or to request missing data. In addition, the quality of primary outcome measures and whether a power calculation was conducted will be assessed. The quality of outcome measurements used will be examined in terms of reliability through internal consistency and test-retest reliability [40]. Only studies using outcome measurements of at least acceptable internal consistency and test-retest reliability (Cronbach’s alpha ≥0.70) will be included [40]. All findings will be summarised within a table to allow easy comparison across studies.

### Data synthesis and analysis

#### Effect size estimates

If possible with available data, a meta-analysis will be conducted using Comprehensive Meta-Analysis Version 2.0 [41]. Post-treatment between group standardised mean difference effect size will be calculated using Hedges’ g from the outcomes relating to depression, anxiety, quality of life and caregiver burden separately. Where multiple time points are reported the longest follow-up time point will be taken ≤6 months. Means and standard deviations of post-outcome measurement scores will be requested from authors if not reported within the paper. Heterogeneity is expected and therefore a random-effect model will be used. In the event that there is no evidence of heterogeneity between studies a fixed-effect model will be selected. The presence of statistically significant heterogeneity will be examined using Cochrane’s test of heterogeneity (Q statistic) and the I^2^ statistic will also be reported to quantify the degree of heterogeneity [42,43]. I^2^ values of heterogeneity will be considered low, moderate or high using cutoffs of 25%, 50% and 75%, respectively [43]. If intention-to-treat data are available these will be used to calculate effect sizes, with completer used when intention-to-treat data are unavailable. With studies that compare two treatment conditions that are eligible for inclusion, comparisons will be analysed separately with the sample size within the control condition halved. Comparisons will be analysed separately with the sample size within the intervention arm halved when two control conditions are included.

#### Funnel asymmetry

Egger’s Test of the Intercept [44] will be used to examine funnel plot asymmetry to investigate possible publication bias and other potential sources of asymmetry (for example, language bias, potential inclusion of small studies with poor methodological rigour, heterogeneity) [44]. Egger’s Test of the Intercept will only be conducted if a minimum of 10 studies are included within the meta-analysis [45]. The trim and fill procedure [46] will be used to calculate an effect size taking into account potential publication bias.

#### Sensitivity analysis

Sensitivity analyses will be undertaken to examine the extent to which results obtained may be influenced by the selective reporting of outcomes. The maximum bias bound approach [47-50] will be adopted with new treatment effect and confidence intervals calculated by adding the bias bound value to the original pooled effect estimate to examine the robustness of findings [48]. Further sensitivity analysis will also be conducted by temporarily dropping from the analysis: small studies (*n* ≤20); unpublished studies; studies with high attrition (≥30%); and studies where outcome measurements of depression and anxiety are reported as primary or secondary outcome measurements to examine whether results remain consistent.

#### Moderator analysis

When number of studies addressing particular moderators permit, moderator analysis will be undertaken to examine intervention components, methodological and participant characteristics of studies associated with effectiveness. Specifically the following moderators will be examined: (1) chronic physical health condition of the care recipient; (2) theoretical framework (for example, cognitive therapy, behaviour therapy); (3) behaviour change techniques used (for example, problem solving, goal setting, relapse prevention); (4) mode of delivery (for example, individual face-to-face, telephone, group); (5) duration of treatment; (6) number of treatment sessions; (7) baseline severity of depression or anxiety; (8) diagnosis of depression or anxiety (yes or no); (9) recruitment setting. Moderators will be examined through subgroup analysis with standardised mean difference effect sizes calculated using Hedges’ g statistic using a random-effects model. Q and I^2^ statistics will also be reported as a measure of heterogeneity. Consistent with other meta-analyses [51,52] subgroup analyses will be considered statistically significant if a *P* value of ≤0.10 is obtained. In the event that there is not enough information in relation to components of interventions to support a meta-analysis [30] a narrative synthesis will be undertaken to summarise these findings.

## Discussion

This review will examine the effectiveness of psychological interventions for informal carers of people with chronic physical health conditions experiencing depression or anxiety. Currently there is no comprehensive review of psychological interventions for informal cares that also systematically examines both the quality of available evidence and intervention components associated with effectiveness. Thereby this review seeks to both examine gaps in the evidence base for future research and also to map intervention components associated with effectiveness. The identification of specific intervention components associated with effectiveness will aid the translation of the existing evidence to the development of new interventions optimising these components. Thus, the mapping of such components is a first step towards developing a psychological treatment for informal carers that maximises the use of behavioural change techniques and delivery factors associated with effectiveness in order to meet objectives within Phase I of the MRC’s guidance [32,33] for developing complex interventions.

## Abbreviations

ASSIA: Applied Social Sciences Index and Abstracts; CBT: Cognitive behavioural therapy; CENTRAL: Cochrane Central Register of Controlled Trials; CINAHL: Cumulative Index to Nursing and Allied Health Literature; CRD: Centre for Reviews and Dissemination; EMBASE: Excerpta Medica DataBase; MeSH: Medical Subject Headings; MRC: Medical Research Council; PROSPERO: International prospective resister of systematic reviews.

## Competing interests

The authors declare that they have no competing interests.

## Authors’ contributions

JW conceived and designed the study protocol and wrote the manuscript. PF contributed to the conception, design and had substantial involvement in the drafting of the manuscript. DR and DL made contributions to the design, critical evaluation of intellectual content and assisted with drafting the manuscript. All authors have approved the final manuscript.

## Supplementary Material

Additional file 1 Ovid MEDLINE Search Strategy. Click here for file

Additional file 2 Data Extraction Form. Click here for file
