# Supplementary material for: Psychological treatments for common mental health problems experienced by informal carers of adults with chronic physical health conditions (Protocol)
Source: Syst Rev. 2013 Jan 31;2:9. doi: 10.1186/2046-4053-2-9 (PMC3599247; doi:10.1186/2046-4053-2-9)
Supplement: Additional file 1 — Ovid MEDLINE Search Strategy. [file 2046-4053-2-9-S1.pdf]

Additional File 1: Ovid MEDLINE Search Strategy

Ovid MEDLINE(R) 1946 to September Week 4 2012

Search Conducted 10th October 2012

1 caregivers/ (18,698)

2 (carer$ or caregiver* or care giver$ or care-giver$ or caregiver-oriented or relative* or family or families or spouse$ or husband$ or wife or wives or partner$ or parent$ or mother$ or father$ or adult$ or dyad$).ti,ab. (2,306,260)

3 exp parents/ (65,102)

4 family/ (57,951)

5 1 or 2 or 3 or 4 (2,341,496)

6 (caring or caregiv$ or care-giv$ or looking after or long term care or caring for or living with or at home or home car$ or family support$ or family car$ or families living or informal car$).ti,ab. (112,905)

7 5 and 6 (60,286)

8 exp Counseling/ (30,688)

9 exp Cognitive Therapy/ (13,525)

10 exp Behavior Therapy/ (47,698)

11 exp Psychotherapy/ or exp Psychotherapy, Multiple/ or exp "Imagery (Psychotherapy)"/ or exp Psychotherapy, Group/ or exp Psychotherapy, Brief/ or exp Psychotherapy, Rational-Emotive/ (141,550)

12 exp Bibliotherapy/ (311)

13 cognitive restructuring.ti,ab. (463)

14 cognitive reframing.ti,ab. (51)

15 behavio$ activation.ti,ab. (911)

16 activity scheduling.ti,ab. (14)

17 problem solving.ti,ab. (10,311)

18 (cCBT or iCBT or computer$ or online or ehealth or e-health or telephone or teletherapy or telehealth or technology).ti,ab. (380,124)

19 (Selfhelp or self help or selfmanag$ or self manage$ or selfadminister$ or self administer$).ti,ab. (29,744)

20 (cognitive or CBT or behavio$ or therap$ or psychotherapy or psychodynamic or counseling or counselling or group or treatment$ or intervention$ or program$ or psychoeducation or psycho education or education or training or manag$ or support$ or psychosocial or rehabilitation).ti,ab. (6,456,654)

21 8 or 9 or 10 or 11 or 12 or 13 or 14 or 15 or 16 or 17 or 18 or 19 or 20 (6,699,948)

22 exp Depression/ (67,563)

23 exp Depressive Disorder/ (74,735)

24 (depression or depressive or depressed or melancholi$ or dysphori$ or dysthymi$ or low mood).ti,ab. (256,054)

25 exp Anxiety/ (49,977)

26 exp Anxiety Disorders/ (61,318)

27 (anxiety or anxious or stress or worry).ti,ab. (447,272)

28 22 or 23 or 24 or 25 or 26 or 27 (715,625)

29 7 and 21 and 28 (8,234)

30 exp Randomized Controlled Trial/ (338,627)

31 exp Clinical Trial/ (699,240)

32 meta-analysis/ (36,848)

33 "review"/ (1,742,840)

34 Random Allocation/ (76,053)

35 (randomi?ed controlled trial$ or RCT or random allocation or randomly allocated or allocated randomly or trial or systematic review or review or meta-analysis).ti,ab. (1,043,085)

36 30 or 31 or 32 or 33 or 34 or 35 (2862890)

37 29 and 36 (2,314)
