# Supplementary material for: Psychological treatments for common mental health problems experienced by informal carers of adults with chronic physical health conditions (Protocol)
Source: Syst Rev. 2013 Jan 31;2:9. doi: 10.1186/2046-4053-2-9 (PMC3599247; doi:10.1186/2046-4053-2-9)
Supplement: Additional file 2 — Data Extraction Form. [file 2046-4053-2-9-S2.docx]

**Additional File 2: Data Extraction Form**

| **Study Identification Features** | | | |
| --- | --- | --- | --- |
| Unique Study Identifier |  | | |
| Title |  | | |
| Authors |  | | |
| Year of Publication |  | | |
| Citation |  | | |
| Publication Type |  | | |
| Country of Origin |  | | |
| Funding Source |  | | |
| **Study Characteristics** | | | |
| Aims and Objectives |  | | |
| Design |  | | |
| Inclusion/Exclusion Criteria |  | | |
| Recruitment |  | | |
| Randomisation | Sequence Generation |  | |
|  | Type |  | |
|  | Allocation Concealment |  | |
|  | Implementation |  | |
| Blinding | Data Collectors |  | |
|  | Data Analysts |  | |
| **Participant Characteristics** | | | |
| Mental Health Condition |  | | |
| Method of Assessment/Diagnosis of Depression |  | | |
| Method of Assessment/Diagnosis of Anxiety |  | | |
| Severity of Depression at Baseline |  | | |
| Severity of Anxiety at Baseline |  | | |
| Age |  | | |
| Ethnicity |  | | |
| Length of time caring |  | | |
| Relationship to Care Recipient |  | | |
| Receipt of Formal Care in the Home |  | | |
| Physical Health Condition of Care Recipient |  | | |
| Age of Care Recipient |  | | |
| Severity of Physical Health Condition of Care Recipient |  | | |
| **Intervention Components** | | | |
| Theoretical Components |  | | |
| Behaviour Change Techniques |  | | |
| Treatment Manual |  | | |
| Measurement of Treatment Integrity |  | | |
| Mode of Delivery |  | | |
| Clinician Delivering Treatment |  | | |
| Training Received by Clinicians |  | | |
| Duration of Treatment |  | | |
| Number of Sessions |  | | |
| Length of Sessions |  | | |
| Treatment Setting |  | | |
| Group size (group based intervention) |  | | |
| Type of Control Condition |  | | |
| **Outcome Measurements** | | | |
| Primary Outcome Measurements |  | | |
| Quality of Primary Outcome Measurements |  | | |
| Length of Follow-up |  | | |
| Secondary Outcome Measurements |  | | |
| Quality of Primary Outcome Measurements |  | | |
| Length of Follow-up |  | | |
| **Statistical Techniques** | | | |
| Power Calculation |  | | |
| Method of Dealing with Missing Data |  | | |
| Baseline Comparability |  | | |
| **Participant Flow** | | | |
| Randomised to Intervention |  | | |
| Randomised to Control |  | | |
| Lost to Follow-Up Intervention^1^ |  | | |
| Lost to Follow-Up Control^1^ |  | | |
| Analysed Intervention^2^ |  | | |
| Analysed Control^2^ |  | | |
| **Results^3^** | | | |
| Intervention | Outcome Measurement | |  |
|  | Pre-Treatment Means | |  |
|  | Pre-Treatment Standard Deviation | |  |
|  | Pre-Treatment Number Analysed | |  |
|  | Post-Treatment Means | |  |
|  | Post-Treatment Standard Deviation | |  |
|  | Post-Treatment Number Analysed | |  |
| Control | Outcome Measurement | |  |
|  | Pre-Treatment Means | |  |
|  | Pre-Treatment Standard Deviation | |  |
|  | Pre-Treatment Number Analysed | |  |
|  | Post-Treatment Means | |  |
|  | Post-Treatment Standard Deviation | |  |
|  | Post-Treatment Number Analysed | |  |
| **Additional Comments** | | | |

^1^Numbers lost to follow-up to be reported at each time point measured; ^2^Numbers analysed to be reported for each time point measured; ^3^Results to be extracted for each primary and secondary outcome measurement at each follow-up time point
